# Supplementary material for: ‘Intelligent’ lockdown, intelligent effects? Results from a survey on gender (in)equality in paid work, the division of childcare and household work, and quality of life among parents in the Netherlands during the Covid-19 lockdown
Source: PLoS One. 2020 Nov 30;15(11):e0242249. doi: 10.1371/journal.pone.0242249 (PMC7703961; doi:10.1371/journal.pone.0242249)
Supplement: S1 Table — A. Descriptive statistics. B. Linear Probability model: Essential occupation. (DOCX) [file pone.0242249.s001.docx]

**S1A Table. Descriptive statistics.**

|  | Mean / Percentage | S.D. |
| --- | --- | --- |
| Male | 44.0% |  |
| Age (in years) | 42.8 | 7.3 |
| *Educational level* |  |  |
| High | 57.4% |  |
| Medium | 30.9% |  |
| Low | 11.7% |  |
| *Number of children* |  |  |
| 1 | 25.1% |  |
| 2 | 47.9% |  |
| 3 | 23.0% |  |
| 4 or more | 4.0% |  |
| *School status of children* |  |  |
| Not at school | 18.1% |  |
| Children in primary school | 34.6% |  |
| Children in high school | 29.8% |  |
| Children in primary & high school | 17.5% |  |
| Essential occupation | 45.9% |  |
| Partner in essential occupation | 32% |  |
| Sector |  |  |
| (semi-)Public | 23.7% |  |
| Private | 44.4% |  |
| Other | 31.9% |  |

**S1B Table. Linear Probability model: essential occupation**

|  | B | S.E |
| --- | --- | --- |
| Intercept | .431*** | .057 |
| Male | -.163*** | .035 |
| Age (centred) | -.004* | .002 |
| (semi)-public sector (private=ref.) | .375*** | .042 |
| Sector unknown | .147*** | .041 |
| Educational level. medium (low=ref.) | .050 | .061 |
| Educational level. high | -.081 | .058 |
| Adjusted R^2^ | 0.146 | |
| N | 748 | |

* p<0.10 **p<0.05 ***p<0.01
