# Supplementary material for: ‘Intelligent’ lockdown, intelligent effects? Results from a survey on gender (in)equality in paid work, the division of childcare and household work, and quality of life among parents in the Netherlands during the Covid-19 lockdown
Source: PLoS One. 2020 Nov 30;15(11):e0242249. doi: 10.1371/journal.pone.0242249 (PMC7703961; doi:10.1371/journal.pone.0242249)
Supplement: S2 Table — A. Distribution of employed respondents by work location. B. Distribution of employed respondents by work location and gender. C. Distribution of employed respondents by work location and educational background. (DOCX) [file pone.0242249.s002.docx]

**S2A Table. Distribution of employed respondents by work location.**

|  | N | % |
| --- | --- | --- |
| Always works from home | 40 | 5.3 |
| Working (almost) all hours from home due to COVID-19 | 281 | 37.6 |
| Working part of hours from home due to COVID-19 | 83 | 11.1 |
| Working (almost) all hours from normal workplace by choice | 51 | 6.8 |
| Working (almost) all hours from normal workplace due to nature of the work | 260 | 34.8 |
| Currently no work tasks | 33 | 4.4 |
| Total | 748 | 100.0 |

**S2B Table. Distribution of employed respondents by work location and gender.**

|  | Fathers | Mothers | Total |
| --- | --- | --- | --- |
| Always working from home | 5.1% | 5.6% | 5.3% |
| Working from home due to the COVID-19 | 38.0% | 37.2% | 37.6% |
| Partly working from home due to the COVID-19 | 12.7% | 9.6% | 11.1% |
| Working outside of home – personal choice | 8.2% | 5.6% | 6.8% |
| Working outside of home – due to occupation | 32.3% | 37% | 34.8% |
| No work | 3.7% | 5.1% | 4.4% |
| N | 353 | 395 | 748 |

**S2C Table. Distribution of employed respondents by work location and educational background.**

|  | Secondary education or less | Post-secondary education | Tertiary education | Total |
| --- | --- | --- | --- | --- |
| Always works from home | 3.9% | 4.7% | 5.9% | 5.3% |
| Working (almost) all hours from home due to COVID-19 | 13.0% | 14.7% | 54.0% | 37.6% |
| Working part of hours from home due to COVID-19 | 5.2% | 9.9% | 12.8% | 11.1% |
| Working (almost) all hours from normal workplace by choice | 5.2% | 5.6% | 7.7% | 6.8% |
| Working (almost) all hours from normal workplace due to nature of the work | 64.9% | 59.1% | 16.6% | 34.8% |
| Currently no work tasks due to corona (furloughed) | 7.8% | 6.0% | 3.0% | 4.4% |
| N | 77 | 232 | 439 | 748 |
