# Supplementary material for: ‘Intelligent’ lockdown, intelligent effects? Results from a survey on gender (in)equality in paid work, the division of childcare and household work, and quality of life among parents in the Netherlands during the Covid-19 lockdown
Source: PLoS One. 2020 Nov 30;15(11):e0242249. doi: 10.1371/journal.pone.0242249 (PMC7703961; doi:10.1371/journal.pone.0242249)
Supplement: S3 Table — A. Working time adaptations by gender. B. Multinomial logistic regression of shifts in working time (ref. = no change to amount of work on normal days off). C. Multinomial logistic regression of shifts in working time (ref. = no change to amount of work in the evenings). D. Multinomial logistic regression of shifts in working time (ref. = no change to amount of work on the weekend). (DOCX) [file pone.0242249.s003.docx]

**S3A Table. Working time adaptations by gender.**

|  | Fathers | Mothers | Total |
| --- | --- | --- | --- |
| Worked (much) less than on a normal workday | 35.1% | 41.3% | 38.3% |
| Worked as much as usual on a normal workday | 50.8% | 39.0% | 44.7% |
| Worked (much) more than on a normal workday | 14.1% | 19.7% | 17.0% |
| N | 333 | 351 | 684 |
| Worked (much) less than on a normal day off | 10.5% | 8.8% | 9.5% |
| Worked as much as on a normal day off | 58.2% | 43.5% | 50.2% |
| Worked (much) more than on a normal day off | 31.4% | 47.7% | 40.2% |
| N | 220 | 262 | 482 |
| Worked (much) less in the evenings | 10.9% | 10.0% | 10.4% |
| Worked as much as usual in the evenings | 51.3% | 46.8% | 49.1% |
| Worked (much) more in the evenings | 37.8% | 43.2% | 40.4% |
| N | 230 | 220 | 450 |
| Worked (much) less in the weekends | 14.3% | 11.4% | 12.8% |
| Worked as much as usual in the weekends | 59.0% | 53.6% | 56.3% |
| Worked (much) more in the weekends | 26.7% | 35.1% | 30.9% |
| N | 210 | 211 | 421 |

**S3B Table. Multinomial logistic regression of shifts in working time (ref. = no change to amount of work on normal days off).**

|  | **Work on normal days off** | | | | | |
| --- | --- | --- | --- | --- | --- | --- |
|  | Less | | | More | | |
|  | B | S.E | OR | B | S.E | OR |
| Male | -0.863* | 0.454 | 0.422 | -0.906*** | 0.326 | 0.404 |
| Essential occupation | -0.500 | 0.487 | 0.607 | 0.447 | 0.308 | 1.563 |
| Male * essential occupation | 1.091 | 0.678 | 2.977 | 0.786* | 0.453 | 2.194 |
| Partner in essential occupation | 0.141 | 0.365 | 1.151 | -0.033 | 0.239 | 0.968 |
| Age (centred) | 0.073** | 0.033 | 1.075 | -0.027 | 0.022 | 0.974 |
| *Sector* |  |  |  |  |  |  |
| Private sector (=ref) |  |  |  |  |  |  |
| (semi-) public sector | -0.433 | 0.461 | 0.648 | -0.116 | 0.279 | 0.890 |
| Sector unknown | -0.252 | 0.393 | 0.777 | -0.359 | 0.269 | 0.699 |
| *Educational level* |  |  |  |  |  |  |
| High | 0.730 | 0.592 | 2.076 | 1.492*** | 0.395 | 4.444 |
| Medium | 0.515 | 0.605 | 1.673 | -0.259 | 0.425 | 0.772 |
| Low (=ref) |  |  |  |  |  |  |
| Number of children | -0.279 | 0.240 | 0.756 | -0.284* | 0.158 | 0.753 |
| *School status of children* |  |  |  |  |  |  |
| No children at school | -0.250 | 0.519 | 0.779 | -0.330 | 0.322 | 0.719 |
| Children at primary school (=ref) |  |  |  |  |  |  |
| Child(ren) at high school | -0.758 | 0.493 | 0.468 | -0.381 | 0.342 | 0.683 |
| Child(ren) at primary school and high school | -0.664 | 0.523 | 0.515 | -0.436 | 0.343 | 0.647 |
| Intercept | -0.747 | 0.823 |  | -0.084 | 0.551 |  |
| Cox & Snell R^2^ | 0.208 | | | | | |
| -2LL (df) | 775.185 (26) | | | | | |
| N | 482 | | | | | |

* p<0.10 **p<0.05 ***p<0.01

**S3C Table. Multinomial logistic regression of shifts in working time (ref. = no change to amount of work in the evenings).**

|  | **Work in the evenings** | | | | | |
| --- | --- | --- | --- | --- | --- | --- |
|  | Less | | | More | | |
|  | B | S.E | OR | B | S.E | OR |
| Male | -0.713 | 0.453 | 0.490 | -0.004 | 0.338 | 0.996 |
| Essential occupation | -0.787 | 0.522 | 0.455 | 0.454 | 0.345 | 1.574 |
| Male * essential occupation | 0.941 | 0.692 | 2.563 | 0.050 | 0.470 | 1.051 |
| Partner in essential occupation | 0.127 | 0.367 | 1.136 | -0.099 | 0.242 | 0.905 |
| Age (centred) | 0.074** | 0.034 | 1.077 | -0.027 | 0.022 | 0.974 |
| *Sector* |  |  |  |  |  |  |
| Private sector (=ref) |  |  |  |  |  |  |
| (semi-) public sector | 0.126 | 0.455 | 1.134 | -0.038 | 0.299 | 0.962 |
| Sector unknown | -0.355 | 0.406 | 0.701 | -0.604** | 0.277 | 0.546 |
| *Educational level* |  |  |  |  |  |  |
| High | 0.408 | 0.559 | 1.505 | 1.647*** | 0.462 | 5.192 |
| Medium | 0.244 | 0.580 | 1.276 | -0.060 | 0.497 | 0.942 |
| Low (=ref) |  |  |  |  |  |  |
| Number of children | -0.049 | 0.229 | 0.952 | -0.201 | 0.159 | 0.818 |
| *School status of children* |  |  |  |  |  |  |
| No children at school | -0.448 | 0.633 | 0.639 | -0.588* | 0.327 | 0.556 |
| Children at primary school (=ref) |  |  |  |  |  |  |
| Child(ren) at high school | -0.240 | 0.501 | 0.787 | -1.027*** | 0.358 | 0.358 |
| Child(ren) at primary school and high school | -0.093 | 0.501 | 0.911 | -0.490 | 0.347 | 0.613 |
| Intercept | -1.176 | 0.803 |  | -0.450 | 0.610 |  |
| Cox & Snell R^2^ | 0.211 | | | | | |
| -2LL (df) | 731.451 (26) | | | | | |
| N | 450 | | | | | |

* p<0.10 **p<0.05 ***p<0.01

**S3D Table. Multinomial logistic regression of shifts in working time (ref. = no change to amount of work on the weekend).**

|  | **Work on weekend** | | | | | |
| --- | --- | --- | --- | --- | --- | --- |
|  | Less | | | More | | |
|  | B | S.E | OR | B | S.E | OR |
| Male | -0.292 | 0.435 | 0.747 | -0.249 | 0.382 | 0.779 |
| Essential occupation | -0.754 | 0.495 | 0.470 | 0.678* | 0.365 | 1.970 |
| Male * essential occupation | 0.489 | 0.653 | 1.630 | 0.140 | 0.500 | 1.150 |
| Partner in essential occupation | 0.195 | 0.348 | 1.215 | 0.161 | 0.253 | 1.175 |
| Age (centred) | 0.033 | 0.032 | 1.034 | -0.012 | 0.023 | 0.988 |
| *Sector* |  |  |  |  |  |  |
| Private sector (=ref) |  |  |  |  |  |  |
| (semi-) public sector | 0.336 | 0.414 | 1.399 | -0.202 | 0.311 | 0.817 |
| Sector unknown | -0.303 | 0.389 | 0.739 | -0.421 | 0.294 | 0.656 |
| *Educational level* |  |  |  |  |  |  |
| High | -0.109 | 0.479 | 0.896 | 1.724*** | 0.568 | 5.609 |
| Medium | -0.183 | 0.504 | 0.833 | 0.431 | 0.600 | 1.539 |
| Low (=ref) |  |  |  |  |  |  |
| Number of children | -0.502** | 0.227 | 0.605 | -0.165 | 0.169 | 0.848 |
| *School status of children* |  |  |  |  |  |  |
| No children at school | -1.168* | 0.615 | 0.311 | -0.687** | 0.347 | 0.503 |
| Children at primary school (=ref) |  |  |  |  |  |  |
| Child(ren) at high school | 0.080 | 0.458 | 1.083 | -0.863** | 0.376 | 0.422 |
| Child(ren) at primary school and high school | 0.140 | 0.494 | 1.150 | -0.355 | 0.357 | 0.701 |
| Intercept | 0.031 | 0.721 |  | -1.284 | 0.722 |  |
| Cox & Snell R^2^ | 0.166 | | | | | |
| -2LL (df) | 707.862 (26) | | | | | |
| N | 421 | | | | | |

* p<0.10 **p<0.05 ***p<0.01
