# Supplementary material for: ‘Intelligent’ lockdown, intelligent effects? Results from a survey on gender (in)equality in paid work, the division of childcare and household work, and quality of life among parents in the Netherlands during the Covid-19 lockdown
Source: PLoS One. 2020 Nov 30;15(11):e0242249. doi: 10.1371/journal.pone.0242249 (PMC7703961; doi:10.1371/journal.pone.0242249)
Supplement: S4 Table — (DOCX) [file pone.0242249.s004.docx]

**S4 Table. Perceived work pressure by gender.**

|  | Fathers | Mothers | Total |
| --- | --- | --- | --- |
| (Much) less work pressure than before the lockdown | 19.2% | 25.4% | 22.5% |
| Same amount of work pressure as before the lockdown | 49.4% | 35.3% | 42.0% |
| (Much) more work pressure than before the lockdown | 31.4% | 39.3% | 35.5% |
| N | 338 | 374 | 712 |
