# Supplementary material for: ‘Intelligent’ lockdown, intelligent effects? Results from a survey on gender (in)equality in paid work, the division of childcare and household work, and quality of life among parents in the Netherlands during the Covid-19 lockdown
Source: PLoS One. 2020 Nov 30;15(11):e0242249. doi: 10.1371/journal.pone.0242249 (PMC7703961; doi:10.1371/journal.pone.0242249)
Supplement: S5 Table — A. Changes in relative share of care tasks. B. Division of care work by gender. (DOCX) [file pone.0242249.s005.docx]

**S5A Table. Changes in relative share of care tasks.**

|  | Fathers | Mothers | Total |
| --- | --- | --- | --- |
| Increase in relative share of care tasks | 21.8% | 12.3% | 16.6% |
| No increase in relative share of care tasks | 78.2% | 87.7% | 83.4% |
| N | 353 | 424 | 777 |
| Decrease in relative share of care tasks | 16.4% | 22.2% | 19.6% |
| No decrease in relative share of care tasks | 83.6% | 77.8% | 80.4% |
| N | 353 | 424 | 777 |

**S5B Table. Division of care work by gender.**

|  | Before the lockdown | | | During the lockdown | | |
| --- | --- | --- | --- | --- | --- | --- |
|  | Fathers | Mothers | Total | Fathers | Mothers | Total |
| Does (much) less than partner | 57.2% | 4.0% | 28.2% | 51.0% | 7.8% | 27.4% |
| Does as much as partner | 36.0% | 31.8% | 33.7% | 38.8% | 32.3% | 35.3% |
| Does (much) more than partner | 6.8% | 64.2% | 38.1% | 10.2% | 59.9% | 37.3% |
| N | 353 | 424 | 777 | 353 | 424 | 777 |
