# Supplementary material for: ‘Intelligent’ lockdown, intelligent effects? Results from a survey on gender (in)equality in paid work, the division of childcare and household work, and quality of life among parents in the Netherlands during the Covid-19 lockdown
Source: PLoS One. 2020 Nov 30;15(11):e0242249. doi: 10.1371/journal.pone.0242249 (PMC7703961; doi:10.1371/journal.pone.0242249)
Supplement: S6 Table — A. Changes in relative share of household tasks by gender. B. Division of household work by gender. (DOCX) [file pone.0242249.s006.docx]

**S6A Table. Changes in relative share of household tasks by gender.**

|  | Fathers | Mothers | Total |
| --- | --- | --- | --- |
| Increase in relative share of household tasks | 17.3% | 7.1% | 11.7% |
| No increase in relative share of household tasks | 82.7% | 92.9% | 88.3% |
| N | 353 | 424 | 777 |
| Decrease in relative share of household tasks | 9.9% | 15.8% | 13.1% |
| No decrease in relative share of household tasks | 90.1% | 84.2% | 86.9% |
| N | 353 | 424 | 777 |

**S6B Table. Division of household work by gender.**

|  | Before the lockdown | | | During the lockdown | | |
| --- | --- | --- | --- | --- | --- | --- |
|  | Fathers | Mothers | Total | Fathers | Mothers | Total |
| Does (much) less than partner | 52.1% | 3.8% | 25.7% | 47.0% | 5.0% | 24.1% |
| Does as much as partner | 39.9% | 26.2% | 32.4% | 43.3% | 30.2% | 36.2% |
| Does (much) more than partner | 7.9% | 70.0% | 41.8% | 9.6% | 64.9% | 39.8% |
| N | 353 | 424 | 777 | 353 | 424 | 777 |
