# Supplementary material for: ‘Intelligent’ lockdown, intelligent effects? Results from a survey on gender (in)equality in paid work, the division of childcare and household work, and quality of life among parents in the Netherlands during the Covid-19 lockdown
Source: PLoS One. 2020 Nov 30;15(11):e0242249. doi: 10.1371/journal.pone.0242249 (PMC7703961; doi:10.1371/journal.pone.0242249)
Supplement: S7 Table — (DOCX) [file pone.0242249.s007.docx]

**S7 Table. Change in leisure time by gender.**

|  | Fathers | Mothers | Total |
| --- | --- | --- | --- |
| (Much) less leisure time | 36.4% | 56.8% | 47.9% |
| Same amount of leisure time | 35.3% | 25.1% | 29.6% |
| (Much) more leisure time | 28.3% | 18.1% | 22.6% |
| N | 371 | 475 | 846 |
