# Supplementary material for: ‘Intelligent’ lockdown, intelligent effects? Results from a survey on gender (in)equality in paid work, the division of childcare and household work, and quality of life among parents in the Netherlands during the Covid-19 lockdown
Source: PLoS One. 2020 Nov 30;15(11):e0242249. doi: 10.1371/journal.pone.0242249 (PMC7703961; doi:10.1371/journal.pone.0242249)
Supplement: S8 Table — (DOCX) [file pone.0242249.s008.docx]

**S8 Table. Ease or difficulty of combining work and care before and during the lockdown.**

|  | Before the lockdown | During the lockdown |
| --- | --- | --- |

|  | Fathers | Mothers | Total | Fathers | Mothers | Total |
| --- | --- | --- | --- | --- | --- | --- |
| Very easy | 17.5% | 14.7% | 16.0% | 13.2% | 12.7% | 12.9% |
| Somewhat easy | 38.7% | 36.8% | 37.7% | 26.4% | 23.9% | 25.0% |
| Neither easy nor difficult | 35.2% | 35.0% | 35.1% | 35.0% | 30.7% | 32.7% |
| Somewhat difficult | 7.2% | 11.4% | 9.4% | 19.8% | 22.8% | 21.4% |
| Very difficult | 1.4% | 2.0% | 1.7% | 5.7% | 9.9% | 7.9% |
| N | 349 | 394 | 743 | 349 | 394 | 743 |
