# Supplementary material for: ‘Intelligent’ lockdown, intelligent effects? Results from a survey on gender (in)equality in paid work, the division of childcare and household work, and quality of life among parents in the Netherlands during the Covid-19 lockdown
Source: PLoS One. 2020 Nov 30;15(11):e0242249. doi: 10.1371/journal.pone.0242249 (PMC7703961; doi:10.1371/journal.pone.0242249)
Supplement: S9 Table — (DOCX) [file pone.0242249.s009.docx]

**S9 Table. Changes in work-life balance.**

|  | N | % |
| --- | --- | --- |
| Work-life balance is more difficult in lockdown | 258 | 34.7 |
| Work-life balance is the same during the lockdown | 417 | 56.1 |
| Work-life balance is easier during the lockdown | 68 | 9.2 |
| Total | 743 | 100 |
