# Supplementary material for: ‘Intelligent’ lockdown, intelligent effects? Results from a survey on gender (in)equality in paid work, the division of childcare and household work, and quality of life among parents in the Netherlands during the Covid-19 lockdown
Source: PLoS One. 2020 Nov 30;15(11):e0242249. doi: 10.1371/journal.pone.0242249 (PMC7703961; doi:10.1371/journal.pone.0242249)
Supplement: S10 Table — (DOCX) [file pone.0242249.s010.docx]

**S10 Table. Never had disagreements with partner on these issues.**

|  | N | % |
| --- | --- | --- |
| Normal workplace | 532 | 78.8 |
| Working from home | 488 | 72.3 |
| Care for children | 330 | 48.9 |
| Household tasks | 270 | 40.0 |
| Leisure time | 379 | 56.1 |
| Total | 748 | 100 |
