# Supplementary material for: ‘Intelligent’ lockdown, intelligent effects? Results from a survey on gender (in)equality in paid work, the division of childcare and household work, and quality of life among parents in the Netherlands during the Covid-19 lockdown
Source: PLoS One. 2020 Nov 30;15(11):e0242249. doi: 10.1371/journal.pone.0242249 (PMC7703961; doi:10.1371/journal.pone.0242249)
Supplement: S11 Table — (DOCX) [file pone.0242249.s011.docx]

**S11 Table. No change in disagreements with partner on these issues.**

|  | N | % |
| --- | --- | --- |
| Normal workplace | 451 | 71.1 |
| Working from home | 386 | 66.6 |
| Care for children | 406 | 61.8 |
| Household tasks | 462 | 70.4 |
| Leisure time | 452 | 70.0 |
| Total | 748 | 100 |
