# Supplementary material for: ‘Intelligent’ lockdown, intelligent effects? Results from a survey on gender (in)equality in paid work, the division of childcare and household work, and quality of life among parents in the Netherlands during the Covid-19 lockdown
Source: PLoS One. 2020 Nov 30;15(11):e0242249. doi: 10.1371/journal.pone.0242249 (PMC7703961; doi:10.1371/journal.pone.0242249)
Supplement: S1 File — (DOCX) [file pone.0242249.s012.docx]

**Supplemental information PlosOne submission**

**Potential editors:**

M Niaz Asadullah

Zélia Maria de Jesus Breda

**Funding statement**

Research material for the Covid19 Gender (In)equality Survey Netherlands (COGIS-NL) study was supported by an [ODISSEI](https://odissei-data.nl/en/2020/04/gender-inequalities-in-times-of-the-covid-19-pandemic/) (Open Data Infrastructure for Social Science and Economic Innovations) grant (ODISSEI, No award number, Dr. Mara A. Yerkes) to collect data during the COVID-19 pandemic. All authors listed on the paper were recipients of the grant (MY, SA, DB, JB, PK, CR, RvdZ, SG). The grant allowed for the collection of data within the existing LISS panel (the Dutch Longitudinal Internet Studies for the Social Sciences). The LISS panel data (including the COGIS-NL data) are collected by CentERdata (Tilburg University, The Netherlands) through its MESS project funded by the Netherlands Organization for Scientific Research. The authors do not receive funding from either CentERdata or the Netherlands Organization for Scientific Research for the purposes of this study. The funder had no role in study design, data collection and analysis, decision to publish, or preparation of the manuscript. CentERdata provided advice regarding study design for the purposes of survey programming; final decisions on study design were the responsibility of the authors. CentERdata had no role in the data analysis, decision to publish, or preparation of the manuscript.

**Ethics**

This study was evaluated and received ethical approval from the Faculty of Social and Behavioural Sciences from Utrecht University. Approval number: 20-269. Ethical approval for data collection rests with CentERdata, the LISS-panel administrator, who requires all respondents to sign a [written, online informed consent form](https://www.lissdata.nl/sites/default/files/bestanden/Informed%20consent%20LISS%20panel%202018%20-%202020.pdf) before participating in the panel.

**Data availability:**

The data underlying the results presented in the study are available from the LISS archive ([www.lissarchive.nl](http://www.lissarchive.nl)). DOI: 10.17026/dans-x3d-e4fb
