# Supplementary material for: ‘Intelligent’ lockdown, intelligent effects? Results from a survey on gender (in)equality in paid work, the division of childcare and household work, and quality of life among parents in the Netherlands during the Covid-19 lockdown
Source: PLoS One. 2020 Nov 30;15(11):e0242249. doi: 10.1371/journal.pone.0242249 (PMC7703961; doi:10.1371/journal.pone.0242249)
Supplement: Attachment — Submitted filename: PONE-D-20-22569_reviewer.pdf [file pone.0242249.s013.pdf]

PLOS ONE

Intelligent lockdown, intelligent effects? The impact of the Dutch COVID-19 ‘intelligent lockdown’ on gendered work and family dynamics among parents

--Manuscript Draft--

**Manuscript Number:**	PONE-D-20-22569
**Article Type:**	Research Article
**Full Title:**	Intelligent lockdown, intelligent effects? The impact of the Dutch COVID-19 ‘intelligent lockdown’ on gendered work and family dynamics among parents
**Short Title:**	Dutch COVID-19 lockdown impact on gendered work and family dynamics
**Corresponding Author:**	Mara A. Yerkes, PhD<br>Universiteit Utrecht<br>Utrecht, NETHERLANDS
**Keywords:**	COVID-19 pandemic; gender inequality; work location; working time adaptation; division of care tasks; division of household tasks; leisure time; quality of life; work-life balance; relationship dynamics; the Netherlands
**Abstract:**	This study examines the impact of the Dutch ‘intelligent lockdown’ during the COVID-19 pandemic on work and family dynamics among parents. This ‘intelligent lockdown’ relied on a combination of restrictive measures and an emphasis on individual responsibility as a means of lessening the spread and health impact of the pandemic. However, the COVID-19 pandemic is more than a public health crisis. Lockdown measures had substantial societal effects, including a significant impact on parents with (young) children. Given gender inequality existent prior to the pandemic, the question arises to what extent the consequences of the lockdown varied for mothers and fathers. Using representative survey data gathered among Dutch parents in April 2020, we explore changes in three areas: paid work, the division of care and household work, and quality of life (leisure, work-life balance, relationship dynamics). Our linear probability and multinomial logistic models demonstrate that the way in which families were impacted by the COVID-19 pandemic reflects a complex gendered reality. We find that gender inequality patterns in the division of paid work, care work, and housework continue to exist. Moreover, the unique situation created by restrictive lockdown measures magnified some inequalities while others were lessened. We find evidence of increased gender inequality in relation to paid work and quality of life, yet a decrease in gender inequality in the division of care and household tasks. During the lockdown, Dutch fathers reported doing more care and household tasks than before. The insights provided here offer key comparative references for understanding the broader impact of lockdown measures on work and family dynamics, as well as quality of life as we move forward in the COVID-19 pandemic.
**Order of Authors:**	Mara A. Yerkes, PhD
	Stéfanie André
	Debby Beckers
	Janna Bessamusca
	Peter Kruyen
	Chantal Remery
	Roos van der Zwan
	Sabine Geurts
**Additional Information:**	
**Question**	**Response**
**Financial Disclosure**<br><br>Enter a financial disclosure statement that	Research material for the Covid19 Gender (In)equality Survey Netherlands (COGIS-NL) study was supported by an ODISSEI (Open Data Infrastructure for Social Science and Economic Innovations) grant to collect data during the COVID-19 pandemic. All

describes the sources of funding for the work included in this submission. Review the submission guidelines for detailed requirements. View published research articles from *PLOS ONE* for specific examples.<br><br>This statement is required for submission and **will appear in the published article** if the submission is accepted. Please make sure it is accurate.<br><br>**Unfunded studies**<br>Enter: *The author(s) received no specific funding for this work.*<br><br>**Funded studies**<br>Enter a statement with the following details:<br>• Initials of the authors who received each award<br>• Grant numbers awarded to each author<br>• The full name of each funder<br>• URL of each funder website<br>• Did the sponsors or funders play any role in the study design, data collection and analysis, decision to publish, or preparation of the manuscript?<br>• **NO** - Include this sentence at the end of your statement: *The funders had no role in study design, data collection and analysis, decision to publish, or preparation of the manuscript.*<br>• **YES** - Specify the role(s) played.<br><br>* typeset	authors listed on the paper were recipients of the grant (MY, SA, DB, JB, PK, CR, RvdZ, SG). The grant allowed for the collection of data within the existing LISS panel (the Dutch Longitudinal Internet Studies for the Social Sciences). The LISS panel data (including the COGIS-NL data) are collected by CentERdata (Tilburg University, The Netherlands) through its MESS project funded by the Netherlands Organization for Scientific Research. The authors do not receive funding from either CentERdata or the Netherlands Organization for Scientific Research for the purposes of this study. The funder had no role in study design, data collection and analysis, decision to publish, or preparation of the manuscript. CentERdata provided advice regarding study design for the purposes of survey programming; final decisions on study design were the responsibility of the authors. CentERdata had no role in the data analysis, decision to publish, or preparation of the manuscript.
**Competing Interests**<br><br>Use the instructions below to enter a competing interest statement for this submission. On behalf of all authors, disclose any competing interests that could be perceived to bias this work—acknowledging all financial support and any other relevant financial or non-financial competing interests.<br><br>This statement **will appear in the published article** if the submission is accepted. Please make sure it is accurate. View published research articles from *PLOS ONE* for specific examples.	The authors have declared that no competing interests exist.

Powered by Editorial Manager® and ProduXion Manager® from Aries Systems Corporation

**NO authors have competing interests**<br><br>Enter: *The authors have declared that no competing interests exist.*<br><br>**Authors with competing interests**<br><br>Enter competing interest details beginning with this statement:<br><br>*I have read the journal's policy and the authors of this manuscript have the following competing interests: [insert competing interests here]*<br><br>* typeset	
**Ethics Statement**<br><br>Enter an ethics statement for this submission. This statement is required if the study involved:<br><br>• Human participants<br>• Human specimens or tissue<br>• Vertebrate animals or cephalopods<br>• Vertebrate embryos or tissues<br>• Field research<br><br>Write "N/A" if the submission does not require an ethics statement.<br><br>General guidance is provided below. Consult the submission guidelines for detailed instructions. **Make sure that all information entered here is included in the Methods section of the manuscript.**	The work presented here has been approved by the Faculty Ethics Review Committee, Faculty of Social and Behavioural Sciences, Utrecht University. Approval number: 20-269.

Powered by Editorial Manager® and ProduXion Manager® from Aries Systems Corporation

**Format for specific study types**<br><br>**Human Subject Research (involving human participants and/or tissue)**<br>• Give the name of the institutional review board or ethics committee that approved the study<br>• Include the approval number and/or a statement indicating approval of this research<br>• Indicate the form of consent obtained (written/oral) or the reason that consent was not obtained (e.g. the data were analyzed anonymously)<br><br>**Animal Research (involving vertebrate animals, embryos or tissues)**<br>• Provide the name of the Institutional Animal Care and Use Committee (IACUC) or other relevant ethics board that reviewed the study protocol, and indicate whether they approved this research or granted a formal waiver of ethical approval<br>• Include an approval number if one was obtained<br>• If the study involved *non-human primates*, add *additional details* about animal welfare and steps taken to ameliorate suffering<br>• If anesthesia, euthanasia, or any kind of animal sacrifice is part of the study, include briefly which substances and/or methods were applied<br><br>**Field Research**<br><br>Include the following details if this study involves the collection of plant, animal, or other materials from a natural setting:<br>• Field permit number<br>• Name of the institution or relevant body that granted permission	
**Data Availability**<br><br>Authors are required to make all data underlying the findings described fully available, without restriction, and from the time of publication. PLOS allows rare exceptions to address legal and ethical concerns. See the PLOS Data Policy and FAQ for detailed information.	No - some restrictions will apply

A Data Availability Statement describing where the data can be found is required at submission. Your answers to this question constitute the Data Availability Statement and **will be published in the article**, if accepted.<br><br>**Important:** Stating 'data available on request from the author' is not sufficient. If your data are only available upon request, select 'No' for the first question and explain your exceptional situation in the text box.<br><br>Do the authors confirm that all data underlying the findings described in their manuscript are fully available without restriction?	
**Describe where the data may be found in full sentences. If you are copying our sample text, replace any instances of XXX with the appropriate details.**<br><br>• If the data are **held or will be held in a public repository**, include URLs, accession numbers or DOIs. If this information will only be available after acceptance, indicate this by ticking the box below. For example: *All XXX files are available from the XXX database (accession number(s) XXX, XXX.).*<br>• If the data are all contained **within the manuscript and/or Supporting Information files**, enter the following: *All relevant data are within the manuscript and its Supporting Information files.*<br>• If neither of these applies but you are able to provide **details of access elsewhere**, with or without limitations, please do so. For example:<br><br>*Data cannot be shared publicly because of [XXX]. Data are available from the XXX Institutional Data Access / Ethics Committee (contact via XXX) for researchers who meet the criteria for access to confidential data.*<br><br>*The data underlying the results presented in the study are available from (include the name of the third party*	The data underlying the results presented in the study will be publicly available from the LISS archive (www.lissdata.nl) following a brief embargo period. The data become available by 1 November 2020 at the latest. We can ensure URLs to the data are available prior to publication.

and contact information or URL).<br>• This text is appropriate if the data are owned by a third party and authors do not have permission to share the data.<br><br>* typeset	
Additional data availability information:	Tick here if the URLs/accession numbers/DOIs will be available only after acceptance of the manuscript for publication so that we can ensure their inclusion before publication.

Powered by Editorial Manager® and ProduXion Manager® from Aries Systems Corporation

Title page Click here to access/download;Manuscript;Title page.docx

Long title: Intelligent lockdown, intelligent effects? The impact of the Dutch COVID-19 'intelligent lockdown' on gendered work and family dynamics among parents

Short title: Dutch COVID-19 lockdown impact on gendered work and family dynamics

Mara A. Yerkes*[1], Stéfanie André[2¶], Debby Beckers[3¶], Janna Besamusca[4¶], Peter Kruyen[2¶], Chantal Remery[5¶], Roos van der Zwan[6¶], Sabine Geurts[3]

[1]Department of Interdisciplinary Social Science, Utrecht University, Utrecht, the Netherlands

[2]Department of Public Administration, Radboud University, Nijmegen, the Netherlands

[3]Behavioural Science Institute, Radboud University, Nijmegen, the Netherlands

[4]Department of Sociology, University of Amsterdam, Amsterdam, the Netherlands

[5]Department of Economics, Utrecht University, Utrecht, the Netherlands

[6]Amsterdam Institute for Labour Studies, University of Amsterdam, Amsterdam, the Netherlands

[¶]These authors contributed equally to this work.

*Corresponding author: M.A.Yerkes@uu.nl (MY)

Cover Manuscript
Click here to access/download;Manuscript;Manuscript_submitted_anonymo

Abstract

This study examines the impact of the Dutch ‘intelligent lockdown’ during the COVID-19 pandemic on work and family dynamics among parents. This ‘intelligent lockdown’ relied on a combination of restrictive measures and an emphasis on individual responsibility as a means of lessening the spread and health impact of the pandemic. However, the COVID-19 pandemic is more than a public health crisis. Lockdown measures had substantial societal effects, including a significant impact on parents with (young) children. Given gender inequality existent prior to the pandemic, the question arises to what extent the consequences of the lockdown varied for mothers and fathers. Using representative survey data gathered among Dutch parents in April 2020, we explore changes in three areas: paid work, the division of care and household work, and quality of life (leisure, work-life balance, relationship dynamics). Our linear probability and multinomial logistic models demonstrate that the way in which families were impacted by the COVID-19 pandemic reflects a complex gendered reality. We find that gender inequality patterns in the division of paid work, care work, and housework continue to exist. Moreover, the unique situation created by restrictive lockdown measures magnified some inequalities while others were lessened. We find evidence of increased gender inequality in relation to paid work and quality of life, yet a decrease in gender inequality in the division of care and household tasks. During the lockdown, Dutch fathers reported doing more care and household tasks than before. The insights provided here offer key comparative references for understanding the broader impact of lockdown measures on work and family dynamics, as well as quality of life as we move forward in the COVID-19 pandemic.

Introduction

Gender inequality in paid work, care work, and household work, is one of the most persistent social problems of the 21st century (1,2). This ongoing inequality significantly affects men and women's quality of life, including leisure time (3,4), work-life balance (5,6), and for those in a relationship, relationship dynamics (7,8). The COVID-19 pandemic and far-reaching measures taken by governments to reduce the spread of the virus have the potential to substantially impact these patterns of gender inequality, especially within families with children. Particularly in the Global North, the pandemic further divided an already gendered labour market. Women are overrepresented in public sector occupations such as health care, education, and childcare (9). In the health care sector alone, three-fourths (76%) of European workers are women (10). Women are similarly overrepresented in health care and community/social service sectors in the US (11) and Australia (12). These commonly underpaid and undervalued occupations became essential during the COVID-19 pandemic; consequently, many mothers continued to work outside the hom[illegible]imultaneously, other female-dominated industries such as retail, accommodation services, and food and beverage service activities have been disproportionately affected by lockdown measures with shops and services in these industries terminating their services. Therefore, women more than men are likely to see the greatest job losses and reductions in working hours during the pandemic and its aftermath (9).

Attempts to slow the spread of COVID-19 has led to office closures and a sudden increase in working from home. In the US, early estimates suggest half of the workforce worked or is working from home full time during the pandemic (13). In Europe, more than one third (37%) of employees were, on average, working from home at the height of the pandemic in the spring of 2020, although this percentage varied across countries, ranging from nearly 20% in Romania to nearly 60% in Finland. Of those who worked from home, one third were

parents with children under the age of 18 (14). The lockdown measures and subsequent increase in working from home had the potential to impact gender dynamics within families because childcare centres and schools were temporarily closed in most countries. Social distancing measures discouraged others, such as grandparents, from caring for (grand)children, therefore alternatives to formal or parental care were limited. As a consequence, care and home-schooling responsibilities shifted fully onto parents.

How do the sudden changes introduced by COVID-19 lockdown measures impact these work and family dynamics? On the one hand, COVID-19 lockdown measures potentially *magnify* structural gender inequalities, for example by reaffirming women's caregiving role (15,16). Initial evidence from countries such as Australia, the UK, the US, and Germany confirm this effect (17–19). In these countries, women spend more time each day on care tasks than men, resulting in an increase on time spent on caregiving tasks during the pandemic. In Australia, Cooper and Mosseri (18) suggest women experience a triple burden during the pandemic: many women face stressful and risky work on the frontlines given their overrepresentation in essential occupations, women are witnessing the greatest losses in jobs and hours, and women's unpaid care tasks at home have increased. On the other hand, COVID-19 lockdown measures have the potential to *reduce* existing social inequalities. Given women's overrepresentation in essential occupations and the concurrent push for non-essential workers to work from home, as well as the absence of care alternatives, families may be forced to (re)negotiate the division of care and/or household work (20). In the US, for example, Carlson, Pepin and Petts (19) show that while women still do more caregiving tasks than men, men are doing more than before the pandemic, and almost no men report doing less.

For a thorough understanding of the impact of the COVID-19 pandemic on gender inequality, more evidence is needed from other countries, as country context is crucial for understanding social processes (21). There is significant cross-national variation in lockdown

measures and supportive social policies in place (22), making country context vital for understanding the short and long-term impact of COVID-19 on gender inequality. The Netherlands presents an interesting case study for furthering this evidence base for two reasons. First, the Netherlands was criticized internationally for its unique and comparatively lenient approach to the COVID-19 lockdown (23). Known as an 'intelligent lockdown' and meant to restrict the spread of the virus but at the same time minimize the economic, social and psychological impact of the pandemic, restrictive measures put in place from 15 March onwards emphasised individual responsibility rather than state enforcement. This approach was viewed as relatively 'soft' compared to other European settings (24). In reality, some measures were strict while others could be considered lenient. Strict measures included the closing of childcare centres, schools and universities; Online classes and homework replaced on-site education to prevent student delays. Emergency care and schooling for children aged 0-12 was available to workers in essential occupations (including care (both youth care and social support), (emergency) childcare, public transport, the food chain (e.g. supermarkets), the transport industry, waste/garbage collection/processing, media and communication, education, emergency services, necessary government processes, farming, and occupations in the funeral industry). Families for whom one parent had an essential occupational while the other did not, were asked to realize their own care arrangements; Emergency care and schooling was intended as a last resort. Further strict lockdown measures included the prohibition of public gatherings and events, which effectively closed down firms and entrepreneurs in the catering and events sector. Bars, restaurants, hairdressers, gyms, and saunas were closed. Other measures, however, were less strict and open to interpretation. Shops and businesses able to maintain social distancing requirements at work could choose to continue operations – either from home or on-site – at their own discretion. Citizens were resolutely asked to stay at home, and all non-essential workers were expected to full-time work from home. Yet public spaces remained

accessible and people were not required to have written authorization for outings, travel or use of public transport (24). However, while families retained the possibility to spend time outside in the company of other household members or one non-family member as long as social distancing was maintained (1.5 meters distance between non-family members), social life effectively came to a standstill and leisure activities were significantly impacted.

Second, the Dutch case is interesting for investigating dynamics in gender inequality because in comparison to other European countries, the Netherlands consistently scores high on gender equality indices in the domains of work, health and knowledge (25). Yet these high scores mask persistent and underlying gender inequality in multiple domains, particularly in relation to paid work and care. For nearly three decades now, the Netherlands has been dominated by its ‘one-and-a-half earner model’: Fathers work full-time, mothers work part-time and spend significantly more time caring for children and doing household tasks (26–28). To date, insufficient pressure existed to challenge the structural inequalities embedded in this Dutch work and family model (29). Against this backdrop, an ‘intelligent lockdown’ has the potential to be a catalyst for change.

As the strategy to move out of lockdown measures unfolds worldwide, social science insights into the impact of the COVID-19 pandemic on gender inequalities in paid work, unpaid work (housework and care), and quality of life are crucial for understanding the effects of the pandemic on societal development and the need for short and long-term policy responses. In this exploratory study, we aim to provide evidence on the immediate effects of COVID-19 measures by studying how parents coped during the intelligent lockdown in the Netherlands. We aim to answer the question: To what extent did the Dutch COVID-19 ‘intelligent lockdown’ increase or decrease gender inequality in work and family dynamics? We contribute to the emerging literature on the societal effects of the COVID-19 pandemic by investigating three main themes for working parents: 1) paid work (work location, working days and times,

perceived work pressure); 2) the division of care and household tasks; and 3) quality of life (leisure, work-life balance, and relationship dynamics). Providing this exploratory evidence will help inform labour market and family policies as well as broader social science debates on gender inequality during pandemics.

Materials and methods

The data used here are the first of four waves of longitudinal data being collected for the COVID Gender (In)equality Study Netherlands (COGIS-NL). Data were collected using a survey module administered to members of the Dutch Longitudinal Internet Studies for the Social Sciences (LISS) panel. The LISS panel is a representativ[illegible]ong-running random sample of individuals registered by the Dutch national statistics office (Statistics Netherlands). The panel consists of approximately 7,000 individuals (4,000 households). The survey was administered by CentERdata, located at Tilburg University, the Netherlands. Fieldwork took place between 13 and 28 April 2020, one month following the start of the intelligent lockdown.

Sample

The requested sample frame included all panel members in a household with at least one member in paid employment and at least one child under the age of 18 living at home. This resulted in a selection of 1,234 panel members. With a response rate of 71.3%, the final sample consisted of 868 respondents in 643 households. A further 16 respondents were excluded from the final analytic sample as their registration in the panel did not match the sampling frame requirements. The final analytic sample consisted of 852 respondents, 748 of whom were in paid work at the time of the survey. Most respondents were in a partnered household; a total of 71 single parents are included in the sample.

Measures

Measures for the survey were tested and developed extensively using a multi-step approach. The first step involved developing an initial set of questions investigating the situation at work and home. These questions were established using our knowledge of the literature. Following, we focused on removing and/or adding questions based on project discussions, time available within the panel (7 minutes), and by comparing our question base with existing LISS modules that can be linked to new data using unique individual and/or household identifiers. In a final step, multiple rounds of pilot testing led to the refinement and finalization of the survey questions. During fielding, all panel respondents are asked if they have comments on the survey; few respondents provided these (either positive or negative) on our questionnaire. The final codebook including all measures is available from the LISS archive (30).

Dependent variables

We explore three themes before *and* during the lockdown for both the respondent and their partner. All measures were self-assessed and reported by one respondent. First, we explore the impact on paid work dynamics by assessing changes to work location, the timing of work, and perceived work pressure. Work location measured whether respondents and/or their partner worked from home, worked at the usual location (required and voluntary), combined working from home with working at the usual location, or were temporarily furloughed (at home because there is currently no work to do). The change in timing of work includes separate analyses for working more or less on normal workdays, normal days off, evenings, and weekends. Lastly, perceived work pressure measured whether respondents experienced more or less work pressure during the lockdown versus prior to the lockdown.

Second, to assess dynamics at home we asked respondents about the division of care and household tasks. We used relative measures, with respondents asked to indicate, relative to their partner, how much housework and caregiving tasks (including home schooling and help with homework) they did before and during the lockdown using a 7-point scale ranging from 'I do nearly everything' to 'My partner does nearly everything'. Third, quality of life was assessed by exploring changes in leisure time, perceived work-life balance, and relationship dynamics (disagreements with partners related to the location of work, the division of care and household tasks, and leisure time). Leisure time was measured on a 5-point scale, ranging from having much less leisure time compared to before the lockdown, to having much more leisure time. Work-life balance was measured using an adapted measure from the European Foundation of Living and Working Conditions (EUROFOUND) Quality of Life survey, measuring how easy or difficult it was for parents to combine paid work with care (including home schooling and homework; measured on a 5-point scale from very easy to very difficult). Lastly, we measured relationship dynamics by asking respondents how often they had disagreements with their partner before and during the pandemic about work location, scheduling of working hours, housework, caring for children, and/or free time.

Independent variables and controls

We are primarily interested in exploring gender differences; our gender measure is included from existing LISS core modules with a binary variable (women=reference category). We also included occupation as an independent variable, measured through self-assessment. Respondents were provided the government list of essential occupations and asked whether they (non-essential=reference category) and/or their partner (partner in non-essential occupation=reference category) worked in an essential occupation. In addition, we controlled for several socio-demographic characteristics, including sector ((semi)-public, private

sector=reference category, sector unknown), age (in years, centred on the grand mean), educational level (low: primary or secondary qualifications=reference category; medium: vocational qualifications; high: tertiary education), number of children, and school status of children under 18 living in the household (measured here as children of primary-school age, children of secondary-school age, children of both primary-school and secondary-school age, and children not attending school=reference category. This category includes both children under school age (0-4) as well as children who have already finished secondary school.

Descriptive data on the sample using these measures can be found in Table S1a. In line with international trends (20), Dutch mothers were overrepresented in essential occupations. Half of all working mothers (56%) were employed in an essential occupation during the lockdown, compared to 34% of fathers. As such, mothers constituted 65% of all essential workers. We note that based on average marginal effects, Dutch fathers were less likely (16 percentage points) to work in an essential occupation than mothers (see Table S1b). As to be expected, most essential workers were employed in the (semi)public sector.

Fifty-seven percent of households and 46% of respondents in the sample reported that at least one parent was employed in an essential occupation. Sixty-one percent of these households reported that one parent was an essential worker while the other was either not in employment or employed in a non-essential occupation, suggesting the other parent might potentially adjust their work and care hours to match those of the essential worker. The remaining households contained families where both parents were employed in essential occupations (28%) and single-parent households (10%). These differences could have important ramifications for the division of paid work, household and care work, and parents' quality of life, as measured through changes in leisure time, work-life balance, and relationship dynamics. We also note that almost all parents in our study (94%) indicated their children were at home either full-time (88%) or part-time (6%) as a consequence of the general closure of

schools and childcare centres during the measurement period. The remaining 6% of children were not at home, and thus in emergency childcare/school.

Methods

In the results section, we report descriptive findings (percentages based on crosstabulations; tables reported in online appendices) supported by multivariate modelling (tables displayed) to control for occupation, partner occupation, age, sector, education, number of children and school status of the children. We use linear regression modelling for continuous outcomes and linear probability modelling (LPM) for binary outcomes (0/1 outcomes). These include the timing of work, the division of care work and housework, leisure time, and relationship dynamics. LPM is a linear regression model on a binary outcome, which is generally preferable to logistic regression for binary outcomes as odds ratios cannot be interpreted as effect measures in mediation or interaction models in logistic regression (31,32). Odds ratios reflect unobserved heterogeneity which can make it difficult to compare across models or groups. LPM yields unbiased and consistent estimates of a variable's average effect on the chance that the outcome occurs. The b-coefficients in LPM can be interpreted as average marginal effects, whereby each coefficient represents a discrete change from the base level holding all other measures constant at their means. Note that average marginal effects report the differences in *percentage points*. Multinomial logistic modelling is used for nominal outcomes (three or more unordered categories in the dependent variable). Multinomial logistic models estimate binary outcomes for all comparisons among the dependent categories, which is not possible using LPM (33,34). We use multinomial logistic regression to explore work location, working time and perceived work pressure, where we report the more easily interpreted odds ratios over logistic coefficients.

Results

Our results show that the Dutch intelligent lockdown, intended to mitigate the health, social and economic impact of the pandemic, significantly impacted Dutch parents' paid work patterns, divisions of care and housework, and quality of life.

The gendered impact on paid work: who works where and when?

Work location

Prior to the lockdown, working from home was possible but not prevalent in the Netherlands (35,36). The COVID-19 pandemic substantially impacted these patterns. Almost half (49%) of the working parents in the sample reported working all (38%) or part (11%) of their hours from home due to the COVID-19 lockdown measures in April 2020 (see Table S2a). However, it would be misleading to suggest everyone worked from home. In our sample, a small share (5%) of working parents indicated they worked most of their hours from home prior to the lockdown, and continued to do so during the lockdown. In addition, substantial shares (42%) of Dutch parents continued to perform work tasks outside the home during the lockdown, perhaps because the Dutch intelligent lockdown allowed businesses and shops to remain open. A small group of respondents (7%) reported being allowed to work from home but chose not to do so. The remainder of the parents not working from home (35% of working parents) indicated they worked on location because their work could not be performed from home. Finally, 4% of parents indicated having a job but being at home without work due to the COVID-19 lockdown measures, suggesting a small proportion of parents was temporarily furloughed.

No clear gender differences emerged from our analyses (see table S2b.). The multinomial logistic regressions with regard to parents who worked all their hours from home,

which controlled for respondent and spouse's employment in essential versus non-essential occupations, age, sector, education, number of children and school status of the children, are shown in Table 1. Work location is primarily associated with respondents' employment in essential versus non-essential occupations. Compared to the reference category of working all hours from home due to COVID-19, respondents in essential occupations were 3.4 times more likely to work part of their hours from home, 8.9 times more likely to work in their normal workplace by choice, and 4.7 times more likely to work in their usual location due to the nature of their work. These effects are the same for mothers and fathers, as indicated by the non-significant interaction terms. Thus, while essential workers were disproportionately women, no additional gender effect was found. A notable exception is that fathers were three times more likely than mothers to work in their normal workplace by choice, compared to working all hours from home, although this effect is only marginally significant.

While the focus here is on gender differences, we note a key finding with our control variables. Variation in work location reflects differences in educational background, which raises questions about the varying impact of the COVID-19 pandemic for lower and higher educated parents. Highly educated parents were substantially more likely to relocate their work to the home: 54% worked entirely from home and 13% performed at least some of their work from home. In contrast, 65% of low educated workers reported working in their usual location because of the nature of their job; 59% of workers with a post-secondary vocational degree did as well (see Table S2c). Specifically, parents with tertiary education were 93.3% less likely than their low educated peers to work in their normal location because their work could not be performed from home compared to working all hours from home.

Table 1. Multinomial logistic regression model: gender differences in work location (ref = working (almost) all hours from home).

	**Working partially from home**			**At normal workplace by choice**			**At normal workplace due to nature of the work**		
	B	S.E	OR	B	S.E	OR	B	S.E	OR
Male	0.413	(0.403)	1.511	1.120*	(0.587)	3.064	0.079	(0.304)	1.082
Essential occupation	1.235***	(0.409)	3.439	2.183***	(0.602)	8.870	1.545***	(0.313)	4.686
Male * essential occupation	0.288	(0.546)	1.333	-0.968	(0.748)	0.380	-0.035	(0.445)	0.965
Partner in essential occupation	-0.291	(0.294)	0.747	0.006	(0.343)	1.006	-0.092	(0.234)	0.912
Age (centred)	0.011	(0.025)	1.011	0.025	(0.030)	1.025	0.014	(0.020)	1.014
Sector									
Private sector (=ref)									
(semi-) public sector	-0.468	(0.332)	0.626	-1.433***	(0.456)	0.239	-0.891***	(0.280)	0.410
Sector unknown	-0.472	(0.348)	0.624	-0.442	(0.393)	0.643	-0.321	(0.266)	0.725
Educational level									
High	-0.496	(0.623)	0.609	-0.896	(0.637)	0.408	-2.701***	(0.390)	0.067
Medium	0.560	(0.662)	1.750	0.097	(0.693)	1.102	-0.145	(0.413)	0.865
Low (=ref)									
Number of children	-0.055	(0.185)	0.947	0.293	(0.217)	1.340	0.110	(0.150)	1.117

School status of children									
No children in school	0.401	(0.374)	1.493	-0.229	(0.507)	0.796	-0.054	(0.322)	0.947
Children in primary school (=ref)									
Child(ren) in secondary school	0.142	(0.412)	1.153	-0.030	(0.473)	0.970	0.183	(0.319)	1.200
Child(ren) in primary and secondary school	0.536	(0.414)	1.709	-0.012	(0.478)	0.988	0.299	(0.328)	1.349
intercept	-1.510	(0.774)		-2.572	(0.921)		0.843	(0.520)	
Cox & Snell R^2	0.332								
-2LL (df)	1290.189 (39)								
N	675								

* $p<0.10$ ** $p<0.05$ *** $p<0.01$

Working time adaptations

As a result of the lockdown and the temporary closure of schools and childcare centres, Dutch parents adapted the days and times at which they worked. Almost two-fifths (38%) of Dutch parents reported working less or much less on their normal workdays compared to the situation prior to COVID-19 (see Table S3a). Substantial shares of parents worked more or much more in the evenings (40%) and during weekends (31%). Mothers were more likely to adapt their working times: While 49% of fathers reported adapting the days on which they normally work during the lockdown, compared to situation prior to the COVID-19 pandemic, 61% of mothers adapted theirs: Forty-one percent worked (much) less on their normal workdays and 20% reported working (much) more on these days. For Dutch fathers, these percentages were 35% and 14% respectively. Mothers were more likely than fathers (48% versus 31%) to work (much) more on days they would normally have off.

Multinomial logistic regressions, controlled for respondent and spouse's employment in essential versus non-essential occupations, age, sector, education, number of children and school status of the children, show that employment in an essential versus non-essential occupation moderates these gendered work dynamics. As Table 2 shows, fathers were 56% less likely to work (much) less on their normal workdays and 44% less likely to work (much) more on their normal workdays. Fathers' lower relative probability of having reduced work during normal working days does not apply to those in essential occupations, as evidenced by the positive interaction term. Mothers in crucial occupations were also less likely to have reduced working on normal working days. The reduction in work during normal workdays was particularly prevalent among mothers employed in non-essential occupations. Fathers' greater tendency to work at normal times is mirrored in their lower relative probability of increasing or decreasing work on normal days off (except for fathers in essential occupations). Net of gender, highly educated parents also experienced more changes to times at which work was

done (Table 2). Compared to low educated parents, highly educated parents were 3.2 times more likely to have reduced work and 3.6 times more likely to have increased work on their normal workdays. The multinomial logistic regressions for further adaptations in working times (working on normal days off, working during evenings and weekends) can be found in Tables S3b-S3d.

Table 2. Multinomial logistic regression model: adaptations in working times (ref.= no change to amount of work on normal workdays).

	**Work on normal workdays**					
	Less			More		
	B	S.E	OR	B	S.E	OR
Male	-0.849***	(0.253)	0.428	-0.589*	(0.356)	0.555
Essential occupation	-0.720***	(0.266)	0.487	0.459	(0.338)	1.583
Male * essential occupation	0.890**	(0.376)	2.435	0.235	(0.477)	1.265
Partner in essential occupation	0.037	(0.198)	1.038	-0.377	(0.258)	0.686
Age (centred)	-0.024	(0.018)	0.976	0.017	(0.022)	1.017
Sector						
Private sector (=ref)						
(semi-) public sector	0.162	(0.241)	1.176	0.122	(0.285)	1.130
Sector unknown	0.330	(0.218)	1.391	-0.203	(0.295)	0.817
Educational level						
High	1.157***	(0.327)	3.179	1.287***	(0.449)	3.623
Medium	-0.118	(0.352)	0.889	0.688	(0.464)	1.989
Low (=ref)						

Number of children	0.010	(0.128)	1.010	-0.041	(0.160)	0.960
School status of children						
No children in school	-0.161	(0.270)	0.851	-0.161	(0.367)	0.851
Children in primary school (=ref)						
Child(ren) in secondary school	-0.192	(0.276)	0.825	0.164	(0.335)	1.178
Child(ren) in primary school and secondary school	-0.639**	(0.287)	0.528	-0.557	(0.371)	0.573
Intercept	-0.215	(0.449)		-1.666	(0.596)	
Cox & Snell R^2	0.153					
-2LL (df)	1251.600 (26)					
N	684					

* p<0.10 **p<0.05 ***p<0.01

More and less work pressure due to the lockdown

The data demonstrate the lockdown led to an increase in work pressure for one-third (36%) of Dutch parents; these parents experienced more work pressure in April than prior to the lockdown (see Table S4). Perceived changes in work pressure differed significantly among mothers and fathers, both in terms of who experienced an increase and who experienced a decrease in work pressure. Remarkably, mothers were significantly more likely than fathers to experience both *more* work pressure (39% versus 31%) as well as *less* work pressure (25% versus 19%) than before the lockdown.

Amongst our control variables (occupation, partner occupation, age, sector, education, number of children and school status of the children), essential occupation matters for

perceived work pressure (see Table 3). Parents in an essential occupation were 2.6 times more likely to report increased work pressure during the lockdown, compared to parents not working in an essential occupation. As with our other analyses, we also find that educational level matters in relation to work pressure. Higher educated parents were more likely to experience changes in work pressure than lower educated parents, with an increased likelihood of experiencing less work pressure as well as more work pressure during the lockdown compared to before.

Table 3. Multinomial logistic regression model: changes in work pressure (ref.= same amount of work pressure as prior to the lockdown).

	Less work pressure			More work pressure		
	B	S.E	OR	B	S.E	OR
Male	-0.630**	(0.214)	0.533	-0.356*	(0.190)	0.700
Age (centred)	-0.001	(0.020)	0.999	-0.012	(0.017)	0.988
Educational level						
High	0.596*	(0.349)	1.815	0.896***	(0.323)	2.450
Medium	0.293	(0.367)	1.340	0.456	(0.337)	1.578
Low (=ref)						
Number of children	-0.089	(0.140)	0.915	-0.114	(0.125)	0.893
School status of children						
No children in school	-0.196	(0.313)	0.822	0.147	(0.270)	1.158
Children in primary school (=ref)						
Child(ren) in secondary school	0.014	(0.296)	1.014	0.051	(0.271)	1.052
Child(ren) in primary and secondary school	-0.158	(0.324)	0.854	0.302	(0.278)	1.352

Essential occupation	0.047	(0.222)	1.048	0.965***	(0.196)	2.624
Partner in essential occupation	0.028	(0.222)	1.028	-0.042	(0.196)	0.959
Sector						
Private sector (=ref)						
(semi-) public sector	-0.123	(0.266)	0.884	-0.244	(0.232)	0.784
Sector unknown	0.033	(0.242)	1.033	-0.209	(0.223)	0.811
Intercept	-0.529	(0.469)		-0.869**	(0.434)	
Cox & Snell R^2	0.082					
-2LL (df)	1415.069 (24)					
N	712					

* p<0.10 **p<0.05 ***p<0.01

The gendered impact on household work and care

Whereas the lockdown appeared to magnify the inequalities in paid work between mothers and fathers, inequality in the division of household work and care declined. Mothers still do the lion's share of care and household tasks, but the gap between mothers and fathers declined some as fathers did more during the lockdown than before.

Caring for children: a changing role for fathers?

The intelligent lockdown led to a general increase in care tasks for roughly one-fifth of parents: Compared to the situation before the lockdown, 17% of parents reported doing a relatively higher share of care tasks during the lockdown (see Table S5a). This share was significantly higher among fathers (22%) than mothers (12%). We also find that a similar percentage of parents (about one-fifth) reported doing less care tasks relative to their partner during the lockdown in comparison to the situation prior to the crisis. Here there are only

negligible differences between mothers and fathers. While more fathers than mothers reported an increase in the relative share of care tasks performed during the lockdown compared to the situation before, the overall division of care between mothers and fathers remains unequal. A majority of mothers (60%) still reported doing (much) more care tasks than their partner during the lockdown (see Table S5b). In contrast, only 10% of fathers reported doing (much) more care tasks than their partner during the lockdown. One-third of parents (34%) reported that prior to the lockdown, care tasks were divided more or less equally, during the lockdown this remained about the same (35%).

Based on the average marginal effects, and controlled for occupation, partner occupation, age, sector, education, number of children and school status of the children, the increase in fathers' relative share of care work is significant (see Table 4). Compared to mothers, fathers had a greater chance (9 percentage points) of reporting an increase in the relative share of care tasks performed during the lockdown than before. In addition to gender, changes in the division of care tasks were clearly related to occupation. Parents who worked in an essential occupation had a significantly smaller chance (13 percentage points) of doing more care tasks during the lockdown than before. The analysis of decreases in the relative shares of care tasks shows the opposite; parents working in essential occupations had a significantly greater chance (14 percentage points) of reporting a decreased relative share of care tasks during the lockdown in comparison to parents working in non-essential occupations. Having a partner in a non-essential occupation did not appear to influence whether respondents reported doing more or less care tasks during the lockdown compared to before.

Table 4. Linear probability model: changes in division of care tasks.

	**Increase in relative share of care tasks compared to before the lockdown**		**Decrease in relative share of care tasks compared to before the lockdown**	
	B	S.E	B	S.E
Male	0.089***	(0.029)	-0.042	(0.032)
Age (centred)	0.001	(0.003)	-0.004	(0.003)
Educational level				
High	0.074	(0.048)	-0.072	(0.052)
Medium	0.043	(0.050)	-0.062	(0.055)
Low				
Number of children	-0.015	(0.020)	0.010	(0.022)
School status of children				
No children in school	-0.030**	(0.041)	-0.038	(0.045)
Children in primary school (=ref.)				
Child(ren) in secondary school	-0.106*	(0.043)	-0.053	(0.047)
Child(ren) in primary and secondary school	-0.067	(0.044)	-0.065	(0.048)
Essential occupation	-0.129***	(0.031)	0.138***	(0.034)

Partner in essential occupation	0.023	(0.030)	-0.029	(0.033)
Sector				
Private (=ref)				
(semi-)public sector	0.038	(0.037)	-0.050	(0.040)
Sector unknown	0.060*	(0.035)	-0.021	(0.038)
Intercept	0.167**	(0.066)	0.266***	(0.073)
Adjusted R^2	0.058		0.031	
N	676			

* p<0.10 **p<0.05 ***p<0.01

Household tasks

For household tasks, there is also limited evidence of change, albeit with smaller effects than by care tasks. A small proportion of parents (12% in total) reported doing more in the household during the lockdown in comparison to before (see Table S6a). Fathers (17%) reported this significantly more often than mothers (7%). A total of 13% of parents reported doing less household work in comparison to before the lockdown, with only negligible differences between fathers and mothers. The division of tasks was and remained unequal, with mothers doing more than fathers. Two-thirds (65%) of all mothers reported doing (much) more household work than their partner during the lockdown (see Table S6b). Among fathers, only 10% indicated they did (much) more than their partner during the lockdown. Similar to the division of care tasks, roughly one third (32%) of parents indicated a more or less equal division of household tasks during the lockdown.

Controlled for occupation, partner occupation, age, sector, education, number of children and school status of the children, the increase in fathers' relative share remained

significant (see Table 5). While mothers continued to do relatively more than fathers, fathers had a significantly greater chance (10 percentage points) than mothers of reporting an increase in their relative share of household work during the lockdown. Considering our control variables, occupation had a larger effect than gender, but only in relation to parents who reported doing less housework during the lockdown. Parents who worked in an essential occupation had a significantly higher chance (11 percentage points) of reporting doing a smaller relative share of housework during the lockdown. Similarly, respondents with a partner working in an essential occupation had a significantly smaller chance (11 percentage points) of reporting doing a smaller relative share of housework during the lockdown.

Table 5. Linear probability model: changes in division of household tasks.

	Does relatively more household tasks		Does relatively less household tasks	
	B	S.E	B	S.E
Male	0.100***	(0.026)	-0.035	(0.028)
Age (centred)	0.000	(0.002)	-0.002	(0.003)
Educational level				
High	0.067	(0.043)	0.003	(0.045)
Medium	0.057	(0.045)	0.013	(0.047)
Low (=ref)				
Number of children	-0.010	(0.018)	-0.029	(0.019)
School status of children				
No children in school	0.022	(0.037)	0.027	(0.039)

Children in primary school (=ref)				
Child(ren) in secondary school	-0.026	(0.038)	0.000	(0.040)
Child(ren) in primary and secondary school	0.041	(0.039)	0.010	(0.041)
Essential occupation	-0.026	(0.028)	0.111***	(0.029)
Partner in essential occupation	0.021	(0.027)	-0.105***	(0.029)
Sector				
Private (=ref)				
(semi)-public sector	0.010	(0.033)	0.041	(0.035)
Sector unknown	0.017	(0.031)	0.007	(0.033)
Intercept	0.031	(0.060)	0.181**	(0.063)
Adjusted R^2	0.021		0.048	
N	676			

* p<0.10 **p<0.05 ***p<0.01

Quality of Life: leisure, work-life balance, and relationship dynamics

The intelligent lockdown impacted the division of paid work, household work, and care work, as well as parent's quality of life in gendered ways.

Mothers faced with less leisure time

For many parents, the lockdown occurred at the expense of leisure time. Almost half (48%) of parents reported having less leisure time (see Table S7). This decline in leisure time is significantly gendered. More than half of the mothers (57%) indicated they had less free time than prior to the lockdown, in comparison to 36% of fathers. These effects remain after controlling for occupation, partner occupation, age, sector, education, number of children, school status of the children, and for furloughed respondents who reported "there is no work for me to do now" (see Table 6). The table shows that parents with an essential occupation faced reduced free time more often compared to parents without an essential occupation. Having children in primary school was also a significant explanatory factor for differences in leisure time: Parents with primary school-aged children were significantly more likely to experience having less free time during the lockdown than before, in comparison to parents with children in secondary school.

Table 6. Linear probability model: changes in leisure time (ref=no change in leisure time).

	**Less leisure time**		**More leisure time**	
	B	S.E	B	S.E
Male	-0.163***	(0.036)	0.092***	(0.031)
Age (centred)	-0.006*	(0.003)	0.002	(0.003)
Educational level				
High	0.241**	(0.058)	-0.013	(0.053)
Medium	0.144***	(0.061)	0.001	(0.051)
Low				

Number of children	0.014	(0.024)	0.008	(0.021)
School status of children				
No children at school	-0.087*	(0.051)	0.026	(0.044)
Children at primary school (=ref.)				
Child(ren) at secondary school	-0.289***	(0.051)	0.198***	(0.045)
Child(ren) at primary and secondary school	-0.037	(0.053)	0.020	(0.047)
Essential occupation	0.100**	(0.037)	-0.011	(0.032)
Partner in essential occupation	0.038	(0.038)	-0.004	(0.033)
Sector				
Private (=ref)				
(semi-)public sector	0.021	(0.045)	-0.047	(0.039)
Sector unknown	-0.016	(0.042)	-0.026	(0.036)
No work for me to do	-0.030	(0.084)	0.341***	(0.072)
Intercept	0.381***	(0.080)	0.114	(0.070)
Adjusted R^2	0.158		0.083	
N	748			

* p<0.10 **p<0.05 ***p<0.01

The intelligent lockdown and perceived work-life balance

Due to the lockdown, more parents faced difficulties in combining work with caregiving responsibilities. The deterioration in work-life balance during the lockdown was equally pronounced between mothers and fathers. Few parents (11%) indicated they found it (somewhat to very) difficult to combine work and care before the lockdown (see Table S8). During the lockdown, however, nearly one-third (29%) of parents perceived the combination

of work and care to be somewhat to very difficult, an increase of 18 percentage points. Looking at changes in work-life balance, one-third (34%) of parents reported having greater difficulty combining work and care during the lockdown than before, and only a small group of parents (9%) perceived the current situation as favourable for their work-life balance (See Table S9).

Controlled for occupation, partner occupation, age, sector, education, number of children and school status of the children, gender is insignificant. Rather than gender differences, educational level and the stage of schooling of children appear to explain variation in perceived work-life balance during the lockdown (see Table 7). Based on the average marginal effects, higher educated parents had a significantly greater chance (16 percentage points) of facing increased difficulty combining paid work and care during the lockdown in comparison to parents with low (primary or secondary) education. This effect is even larger for parents with children of primary school age. Having primary school aged children significantly increased the chance of experiencing difficulty combining work and care (by 28 percentage points).

Table 7. Linear probability model: change in work-life balance.

	**Easier to combine work and care**		**More difficult to combine work and care**	
	B	S.E	B	S.E
Male	-0.032	(0.022)	-0.034	(0.036)
Age (centred)	0.000	(0.002)	-0.002	(0.003)
Educational level				
High	0.002	(0.036)	0.163***	(0.058)
Medium	-0.009	(0.038)	0.079	(0.061)

Low				
Number of children	-0.016	(0.015)	-0.001	(0.024)
School status of children				
No children at school	0.003	(0.032)	-0.068	(0.050)
Children at primary school (=ref.)				
Child(ren) at secondary school	0.068**	(0.032)	-0.284***	(0.051)
Child(ren) at primary and secondary school	0.055*	(0.033)	-0.081	(0.053)
Essential occupation	-0.023	(0.023)	0.045	(0.037)
Partner in essential occupation	-0.019	(0.024)	0.048	(0.037)
Sector				
Private (=ref)				
(semi-)public sector	0.024	(0.028)	-0.004	(0.044)
Sector unknown	0.063**	(0.026)	-0.021	(0.041)
Intercept	0.105**	(0.050)	0.324***	(0.079)
Adjusted R^2	0.010		0.080	
N	748			

* p<0.10 **p<0.05 ***p<0.01

Relationship dynamics: marginal increases in disagreements between partners

Our final measure of quality of life relates to relationship dynamics: The extent to which partners experienced disagreements about the location of work, the division of care or household tasks, or the amount of free time. Most parents indicated that prior to the lockdown, they never had disagreements on most of these topics, including where they or their partner

worked (working at the normal work location (79%); working from home (72%)), or the amount of free time (56%; see Table S10). However, prior to the lockdown, about half of parents experienced monthly (or more frequent) disagreements about the division of care tasks (51%), and 60% quarrelled about household tasks. The lockdown did not change this situation for most parents. A majority of parents (between 62% and 71%, depending on the measure) reported no change in the prevalence of disagreements in all areas during the lockdown (the normal work location, working from home, caring for children, household chores, and leisure time; see Table S11).

These relationship dynamics were the same for mothers and fathers, even after controlling for occupation, partner occupation, age, sector, education, number of children and school status of the children. The only significant increase in disagreements was reported amongst a proportion of parents in relation to the division of care tasks. One-fifth of parents reported an increase in disagreements about the division of care tasks, with no differences related to gender (see Table 8). However, parents with children in primary school had a higher chance of reporting an increase in disagreements about the division of care tasks (13 percentage points) than parents with children in secondary school.

Table 8. Linear probability model: change in disagreements about division of childcare.

	Less disagreement about childcare tasks		More disagreement about childcare tasks	
	B	S.E	B	S.E
Male	-0.024	(0.031)	-0.021	(0.033)
Age (centred)	0.002	(0.003)	-0.009***	(0.003)
Educational level				
High	-0.169***	(0.050)	0.168***	(0.054)

Medium	-0.138***	(0.053)	0.076	(0.057)
Low				
Number of children	-0.005	(0.020)	-0.034	(0.022)
School status of children				
No children at school	0.011	(0.043)	-0.104**	(0.046)
Children at primary school (=ref.)				
Child(ren) at secondary school	0.007	(0.045)	-0.128***	(0.048)
Child(ren) at primary and secondary school	-0.024	(0.045)	0.003	(0.049)
Essential occupation	0.034	(0.032)	0.005	(0.035)
Partner in essential occupation	-0.025	(0.032)	0.006	(0.034)
Sector				
Private (=ref)				
(semi-)public sector	-0.080**	(0.038)	-0.043	(0.041)
Sector unknown	-0.058	(0.036)	0.067*	(0.038)
Intercept	0.360***	(0.069)	0.220***	(0.074)
Adjusted R^2	0.018		0.081	
N	748			

* p<0.10 **p<0.05 ***p<0.01

Discussion and conclusion

The lockdown measures taken in the spring of 2020 to curb the spread of COVID-19 in the Netherlands clearly impacted Dutch parents. Many parents experienced significant changes in paid work (including where and when they worked, and subjective work pressure), the

division of household and care tasks, and quality of life (the amount of leisure time, as well as the perceived ease or difficulty of combining work and care tasks and negotiating about these tasks with their partner).

Our study indicates that this impact of the COVID-19 lockdown measures on Dutch parents was gendered. The emergence of (and in some cases worsening) of gender inequalities is evident across multiple areas. Mothers adapted the times at which they work, more so than fathers. Mothers not in essential occupations adapted their working times even more, and were more likely to experience increased work pressure during the lockdown. At home, the division of household and care tasks remained unequal. More mothers than fathers reported doing relatively more housework and care tasks than their partner, before the lockdown as well as during the lockdown. We do find limited evidence of a reduction in gender inequality in the division of care and household tasks, however. While mothers continued doing more household and caregiving tasks than fathers, the gap decreased somewhat as fathers reported doing (somewhat) more during lockdown. We do not find that mothers experienced a more pronounced overall decline in the quality of life in comparison to fathers. Leisure time, an important indicator of quality of life, dramatically decreased during the lockdown for mothers, yet mothers and fathers equally experienced a worsening of work-life balance and an increase in disagreements about the negotiation of the division of housework and care tasks.

The changes in caregiving and household tasks provide some reason for optimism. While mothers still do the lion’s share of caregiving and household tasks, the increased involvement of fathers in these tasks during the lockdown offers an opportunity and potential catalyst for further erosion of traditional role patterns in the division of household and care tasks. Prior to the lockdown, Dutch couples routinely reported wanting an equal division of work and care, yet fathers remained more likely to participate in paid employment, with mothers taking on a higher caregiving burden (28). The question now remains whether these

changes towards a more equal division of care and household tasks will be sustained as the Netherlands relaxes lockdown measures and returns to a ‘new normal’.

Alongside the gender differences explored here, our study also raises questions about the effects of working in an essential occupation as well as working from home, especially with regard to quality of life. We found that parents working in an essential occupation experienced the highest levels of work pressure during the lockdown, which can result in health-related risks, such as burnout. These workers were also less likely to work from home. Workers in essential occupations, more often mothers than fathers, and the lower educated, largely continued to perform work in their usual workplace. As a result, these workers face larger risks of exposure to the COVID-19 virus, especially if they work in care occupations, use public transport to get to work, or interact closely with colleagues.

Meanwhile, our study also indicates that working from home, largely heralded in the media and by politicians as ‘the new normal’, is reserved primarily for highly educated parents working in public sector, non-essential occupations. These parents face potentially larger risks related to the blurring of work and family time, as evidenced by their greater tendency to deviate from their normal work days and times. Future research must consider the long-term effects of (not) working from home for mothers and fathers, especially with regard to their health and quality of life. In particular, the future world of work is expected to enable greater accessibility of working from home. Based on our findings, we can speculate about what this might imply for gender inequality. On the one hand, it could be that more opportunities to work from home (and increased flexibility in working hours) would enable mothers to increase their participation in paid work. On the other hand, unfavourable side effects can be expected when we consider our finding that fathers more often chose to go to the workplace during lockdown even when they could work from home, whereas mothers more often worked from home. In the future, such differences may result in gender inequality with mothers possibly becoming

‘online’ workers, with potentially fewer career and networking opportunities, and a greater blurring of work and family life. Although working from home can be advantageous to some workers, employers need to anticipate and monitor potential for unfavourable side effects. Future research needs to closely follow the effects of the more intensive forms of working from home that have been introduced in many countries as a result of the pandemic.

The gender and educational inequalities (a proxy for class inequalities) exacerbated by or brought about by the lockdown measures are also cause for concern. While mothers’ greater adaptability need not be problematic in and of itself, the larger decrease in free time among mothers and the increased perception of work pressure among mothers in comparison to fathers, is. People with insufficient free time experience a poorer quality of life as well as reduced health and well-being (3). Greater perceived work pressure and difficulty combining work and care can also have long-term negative effects. Employees who experience high stress, high work load and time constraints for a long period of time are less productive, are more likely to suffer from burnout complaints and are more likely to switch jobs compared to employees who are feeling well (37–39). This is especially true when employees have insufficient time to recover from work stress on a day-to-day basis (40). High levels of perceived work pressure, both before and during the lockdown, among mothers and among workers in occupations considered to be essential for society is therefore worrisome. Particularly as mothers also experience insufficient leisure time, which can be a sign of insufficient recovery. These concerns are even greater when considered in relation to growing staff shortages in some of the occupational groups where mothers are overrepresented (e.g., care and education).

We note a number of limitations of our exploratory study, primarily related to data availability. Data on the age of the youngest child are unavailable. The distinction made here based on the phase of schooling addresses this issue only partially. Future research will need

to account for the age of children, particularly in order to distinguish the pressure of care tasks for children younger than school age versus children living at home, but no longer attending school. In addition, data on working hours would be useful, particularly in the Dutch case; mothers working part-time might be the ones adapting their work situation more than full-time working mothers. Lastly, while we have been able to explore key changes in paid work, and the division of care and household tasks, more data on how parents experienced the changed division of care and household work is needed. Do fathers who took on greater care or household tasks during the lockdown enjoy these new roles? Do mothers like these new divisions, or do some feelings of discontent arise? Questions such as these become crucial for understanding the potential long-term sustainability of such improvements in gender equality.

The strengths of our study – a representative study among Dutch parents with a high response rate, conducted in the midst of an unprecedented lockdown – outweigh these limitations. The findings here show evidence of an international trend in which inequalities along gender and class lines are worsening, although cross-national differences and some signs of declining inequality clearly remain. Job losses have been exponential, but certainly not the same across countries (17). Women appear to be impacted more by job losses in several countries, including the Netherlands (41), and also in the US, the UK, and Australia. The latter three countries have historically less generous social policies in place to mitigate the consequences of being unable to work (17,18). Such variation in policy schemes available to support the temporary furloughing of employees may help explain the differing effects of lockdown measures in the long run (17,22). With or without government support, the sectors in which women work have been harder hit by the lockdown (e.g. industries such as retail, accommodation services, and food and beverage service activities) and it is expected that these effects will be felt far into the future (42).

Studies such as ours demonstrate the need to consider the societal impact of lockdown measures on parents and families. In addition, while the closure of schools and childcare centres led to greater attention to the needs of parents and families, there is clearly a necessity to consider the societal impact on *all* workers affected by the lockdown measures. The class effects found here extend beyond parents. Workers on temporary contracts and the self-employed faced and continue to face significant work and income insecurity (14,17). Workers in essential occupations experienced significant work pressure. Simultaneously, social distancing requirements caused social networks to fall away for almost everyone. The impact of these measures differs across individuals, with young people experiencing much higher levels of loneliness during the lockdown than before (14).

It remains unclear whether and to what extent new lockdown measures will be implemented in the coming months. If anything, the COVID-19 virus has shown itself to be highly unpredictable. The measures taken to reduce its spread will likely mirror this situation. Our study suggests, however, that in potential future lockdowns, policy and research attention should be paid to gendered and classed differences in free time and work pressure to mitigate the adverse impact on health and wellbeing of parents, especially among mothers. Should schools need to be closed during future lockdowns, greater support for parents, and more independent assignments for (younger) children, may help to mitigate the pressure parents feel when negotiating care tasks with their partner. In short, the societal impact of the lockdown measures is great. While scientific and policy attention is logically focused on the health risks associated with the COVID-19 virus, the societal implications of attempts to stop its spread must not be forgotten.

Acknowledgements

We are grateful to ODISSEI (Open Data Infrastructure for Social Science and Economic Innovations) for supporting data collection for this Covid19 Gender (In)equality Survey Netherlands (COGIS-NL) study. We are also grateful to Paul de Beer (University of Amsterdam) for his contribution to the initial study and to Joost Leenen of CentERdata (Tilburg University, The Netherlands) for programming the survey.

References

1. Abendroth A-K, Huffman M, Treas J. The Parity Penalty in Life Course Perspective: Motherhood and Occupational Status in 13 European Countries. Am Sociol Rev [Internet]. 2014;79(5):993–1014. Available from: http://asr.sagepub.com/cgi/doi/10.1177/0003122414545986
2. Yerkes MA, Martin B, Baxter J, Rose J. An unsettled bargain? Mothers? perceptions of justice and fairness in paid work. J Sociol [Internet]. 2017 Jun 13 [cited 2017 Jun 14];53(2):476–91. Available from: http://journals.sagepub.com/doi/10.1177/1440783317696361
3. Yerkes MA, Roeters A, Baxter J. Gender differences in the quality of leisure: A cross-national comparison. Community Work Fam [Internet]. 2018 Oct 19;1–18. Available from: https://www.tandfonline.com/doi/full/10.1080/13668803.2018.1528968
4. Bittman M, Wajcman J. The Rush Hour: The Character of Leisure Time and Gender Equity. Soc Forces [Internet]. 2000 Sep [cited 2016 Sep 25];79(1):165–89. Available from: http://www.jstor.org/stable/2675568?origin=crossref
5. Beham B, Drobnič S, Präg P, Baierl A, Eckner J. Part-time work and gender inequality in Europe: a comparative analysis of satisfaction with work-life balance. Eur Soc [Internet]. 2018 [cited 2018 Aug 11];1–25. Available from: http://www.tandfonline.com/action/journalInformation?journalCode=reus20
6. Milkie MA, Kendig SM, Nomaguchi KM, Denny KE. Time With Children, Children's Well-Being, and Work-Family Balance Among Employed Parents. J Marriage Fam [Internet]. 2010 Sep 29 [cited 2018 May 4];72(5):1329–43. Available from: http://doi.wiley.com/10.1111/j.1741-3737.2010.00768.x
7. Blom N, Hewitt B. Becoming a Female- Breadwinner Household in Australia: Changes in Relationship Satisfaction. J Marriage Fam [Internet]. 2020 Aug 26 [cited

2020 Jul 20];82(4):1340–57. Available from: https://onlinelibrary.wiley.com/doi/abs/10.1111/jomf.12653

8. Klumb P, Hoppmann C, Staats M. Division of Labor in German Dual-Earner Families: Testing Equity Theoretical Hypotheses. J Marriage Fam. 2006;68(4):870–82.

9. Queisser M, Adema W, Clarke C. COVID-19, employment and women in OECD countries. 2020.

10. EIGE. Frontline Workers [Internet]. Vilnius; 2020. Available from: https://eige.europa.eu/covid-19-and-gender-equality/frontline-workers

11. US Bureau of Labor Statistics. Employed persons by detailed occupation, sex, race, and Hispanic or Latino ethnicity [Internet]. Labor Force Statistics from the Current Population Survey. 2020 [cited 2020 Jul 20]. Available from: https://www.bls.gov/cps/cpsaat11.htm

12. Australian Bureau of Statistics. Employment by industry [Internet]. Labour market information portal. 2020 [cited 2020 Jul 20]. Available from: https://lmip.gov.au/default.aspx?LMIP/Downloads/EmploymentRegion

13. Brynjolfsson E, Horton JJ, Ozimek A, Rock D, Sharma G, TuYe H-Y. COVID-19 and Remote Work: An early look at US data [Internet]. NBER Working Paper Series. Cambridge, MA; 2020. Report No.: 27344. Available from: https://www.nber.org/papers/w27344.pdf

14. Eurofound. Living , working and COVID-19 First findings – April 2020 [Internet]. Dublin; 2020. Available from: https://www.eurofound.europa.eu/sites/default/files/ef_publication/field_ef_document/ef20058en.pdf

15. Wenham C, Smith J, Morgan R, Group W. Comment COVID-19: the gendered impacts of the outbreak. Lancet [Internet]. 2020 Mar 14 [cited 2020 Mar

20];395(10227):846–8. Available from: https://doi.org/10.1016/S0140-6736
16. Lewis H. The Coronavirus Is a Disaster for Feminism. The Atlantic [Internet]. 2020 [cited 2020 Mar 20]; Available from: https://www.theatlantic.com/international/archive/2020/03/feminism-womens-rights-coronavirus-covid19/608302/
17. Adams-Prassl A, Boneva T, Golin M, Rauh C. Inequality in the impact of the coronavirus shock: new survey evidence for the UK [Internet]. Bonn; 2020. (IZA DP). Report No.: No. 13183. Available from: https://www.inet.econ.cam.ac.uk/working-paper-pdfs/wp2010.pdf%0Ahttps://www.cam.ac.uk/research/news/younger-workers-hit-harder-by-coronavirus-economic-shock-in-uk-and-us
18. Cooper R, Mosseri S. Pandemic has impacted on women most significantly. The Sydney Morning Herald. 2020 Jun 5;
19. Carlson D, Petts R, Pepin J. US Couples' Divisions of Housework and Childcare during COVID-19 Pandemic [Internet]. 2020 [cited 2020 Jul 20]. Available from: https://osf.io/preprints/socarxiv/jy8fn/
20. King T, Hewitt B, Crammond B, Sutherland G, Maheen H, Kavanagh A. Reordering gender systems: can COVID-19 lead to improved gender equality and health? Lancet [Internet]. 2020 [cited 2020 Jun 25]; Available from: https://doi.org/10.1016/S0140-6736
21. Ciccia R, Javornik J. Methodological Challenges for Comparative Welfare State Research: Capturing Intra-Country Variation in Cross-National Analyses. J Comp Policy Anal Res Pract [Internet]. 2019;21(1):1–8. Available from: https://www.tandfonline.com/doi/full/10.1080/13876988.2018.1551598
22. ILO. COVID-19 and the world of work. Country policy responses [Internet]. 2020 [cited 2020 Jun 28]. Available from:

https://www.ilo.org/global/topics/coronavirus/country-responses/lang--en/index.htm

23. BBC News. Coronavirus: Why Dutch lockdown may be a high-risk strategy - BBC News [Internet]. 2020 [cited 2020 Jun 18]. Available from: https://www.bbc.com/news/world-europe-52135814

24. De Voogd C. Europe Versus Coronavirus - The 'Intelligent Lockdown' Gamble of the Netherlands | Institut Montaigne [Internet]. Institut Montaigne Blogs. 2020 [cited 2020 Jul 20]. Available from: https://www.institutmontaigne.org/en/blog/europe-versus-coronavirus-intelligent-lockdown-gamble-netherlands

25. EIGE. Gender equality index 2019: Netherlands [Internet]. Vilnius; 2019 [cited 2020 Jun 18]. Available from: https://eige.europa.eu/publications/gender-equality-index-2019-netherlands

26. Yerkes MA, Hewitt B. Part-time work strategies of women and men of childbearing age in the Netherlands and Australia. In: Nicolaisen H, Kavli HC, Jensen RS, editors. Dualisation of part-time work: the development of labour market insiders and outsiders. Bristol: Policy Press; 2019.

27. Visser J. The first part-time economy in the world: a model to be followed? J Eur Soc Policy. 2002;12(1):23–42.

28. Portegijs W, van den Brakel M. Emancipatiemonitor 2018 [Internet]. Cornelissen S, editor. Emancipatiemonitor: 2018. Den Haag: Centraal Bureau voor de Statistiek en Sociaal en Cultureel Planbureau; 2018 [cited 2019 May 28]. Available from: https://digital.scp.nl/emancipatiemonitor2018/

29. Ministerie van Financien. IBO Deeltijd. De(el)tijd zal het leren: Van analyse naar beleid over deeltijd. Den Haag; 2020.

30. Scherpenzeel AC, Das M. "True" Longitudinal and Probability-Based Internet Panels: Evidence From the Netherlands. In: Das M, Ester P, Kaczmirek L, editors. Social and

Behavioral Research and the Internet: Advances in Applied Methods and Research Strategies [Internet]. Boca Raton: Taylor & Francis; 2010. p. 77–104. Available from: www.lissdata.nl

31. Mood C. Logistic Regression: Why We Cannot Do What We Think We Can Do, and What We Can Do About It. Eur Sociol Rev [Internet]. 2010 Feb 1 [cited 2018 Mar 21];26(1):67–82. Available from: https://academic.oup.com/esr/article-lookup/doi/10.1093/esr/jcp006

32. Hellevik O. Linear versus logistic regression when the dependent variable is a dichotomy. Qual Quant. 2009;43(1):59–74.

33. Borooah VK. Logit and probit : ordered and multinomial models. Thousand Oaks, CA: Sage Publications; 2002. vi, 97.

34. Fullerton AS. Logit and Probit: Binary and Multinomial Choice Models. In: SAGE Research Methods Foundations. SAGE Publications Ltd; 2020.

35. Josten E (Edith JC, Merens JGF (Johanna GF, Portegijs W (Willy), Josten E (Edith JC, Voogd-Hamelink M de (A. M. Arbeidsmarkt in kaart: wel- en niet-werkenden. Arbeidsmarkt in kaart : Wel- en niet-werkenden – editie 1. Den Haag: Sociaal en Cultureel Planbureau; 2019 Jun.

36. van Echtelt P, Putman L, de Voogd-Hamelink M. Arbeidsmarkt in kaart: Werkgevers - editie 2. Arbeidsmarkt in kaart: Werkgevers - editie 2. Den Haag: Sociaal en Cultureel Planbureau; 2019.

37. Lopes H, Lagoa S, Calapez T. Work autonomy, work pressure, and job satisfaction: An analysis of European Union countries. Econ Labour Relations Rev. 2014;25(2):306–26.

38. Lu Y, Hu X-M, Huang X-L, Zhuang X-D, Guo P, Feng L-F, et al. The relationship between job satisfaction, work stress, work-family conflict, and turnover intention

among physicians in Guangdong, China: a cross-sectional study. BMJ Open [Internet]. 2017 [cited 2020 May 25];7. Available from: http://dx.doi.org/10.1136/bmjopen-2016-014894

39. Riedl EM, Thomas J. The moderating role of work pressure on the relationships between emotional demands and tension, exhaustion, and work engagement: an experience sampling study among nurses. Eur J Work Organ Psychol [Internet]. 2019;28(3):414–29. Available from: https://doi.org/10.1080/1359432X.2019.1588251

40. Geurts SAE, Sonnentag S. Recovery as an explanatory mechanism in the relation between acute stress reactions and chronic health impairment. Scand J Work Environ Heal. 2006;32(6):482–92.

41. Jongen E, Verstraten P, Siflinger B. Arbeidsmarkt: Historische daling werkzame personen, maar internationaal gezien beperkt [Internet]. Den Haag; 2020 [cited 2020 Jul 20]. Available from: https://www.cpb.nl/sites/default/files/omnidownload/CPB-coronapublicatie-juni2020-Internationale-vergelijking-arbeidsmarkt.pdf

42. Alon TM, Doepke M, Rumsey JO, Tertilt M. The Impact of Covid-19 on Gender Equality. Cambridge, MA; 2020. (NBER Working Paper). Report No.: 26947.

Supporting information captions

S1a Table. Descriptive statistics.

S1b Table. Linear Probability model: essential occupation

S2a Table. Distribution of employed respondents by work location.

S2b Table. Distribution of employed respondents by work location and gender.

S2c Table. Distribution of employed respondents by work location and educational background.

S3a Table. Working time adaptations by gender.

S3b Table. Multinomial logistic regression of shifts in working time (ref. = no change to amount of work on normal days off).

S3c Table. Multinomial logistic regression of shifts in working time (ref. = no change to amount of work in the evenings).

S3d Table. Multinomial logistic regression of shifts in working time (ref. = no change to amount of work on the weekend).

S4 Table. Perceived work pressure by gender.

S5a Table. Changes in relative share of care tasks.

S5b Table. Division of care work by gender.

S6a Table. Changes in relative share of household tasks by gender.

S6b Table. Division of household work by gender.

S7 Table. Change in leisure time by gender.

S8 Table. Ease or difficulty of combining work and care before and during the lockdown.

S9 Table. Changes in work-life balance.

S10 Table. Never had disagreements with partner on these issues.

S11 Table. No change in disagreements with partner on these issues.

Click here to access/download

Supporting Information - Compressed/ZIP File Archive

Supplemental information files.zip
