# Supplementary material for: ‘Intelligent’ lockdown, intelligent effects? Results from a survey on gender (in)equality in paid work, the division of childcare and household work, and quality of life among parents in the Netherlands during the Covid-19 lockdown
Source: PLoS One. 2020 Nov 30;15(11):e0242249. doi: 10.1371/journal.pone.0242249 (PMC7703961; doi:10.1371/journal.pone.0242249)
Supplement: Attachment — Submitted filename: Reviewer comment 15082020.pdf [file pone.0242249.s014.pdf]

Reviewer comment

a) There is a big confounder of having alternative care personal in the family, as this will affect both individual as well as relationship with partner and until multivariate regression looks into this the causality cannot be established
b) The LISS data needs to explained as there can be bias due to the dataset itself
c) Difference of pre and post covid is not being established through background as well as result section, some relationship demonstration will be extremely important.
